# Game analysis on the evolution of COVID-19 epidemic under the prevention and control measures of the government

**DOI:** 10.1371/journal.pone.0240961

**Published:** 2020-10-23

**Authors:** Jinyu Wei, Li Wang, Xin Yang

**Affiliations:** 1 School of Management, Tianjin University of Technology, Tianjin, China; 2 Zhonghuan Information College Tianjin University of Technology, Tianjin, China; Wenzhou University, CHINA

## Abstract

In this paper, the interaction strategies and the evolutionary game analysis of the actions taken by the government and the public in the early days of the epidemic are incorporated into the natural transmission mechanism model of the epidemic, and then the transmission frequency equations of COVID-19 epidemic is established. According to the cumulative confirmed cases of COVID-19 in the UK and China, the upper limit of the spread of COVID-19 in different evolutionary scenarios is set. Using SPSS to perform logistic curve fitting, the frequency fitting equations of cumulative confirmed cases under different evolution scenarios are obtained respectively. The analysis result shows that the emergency response strategy adopted by the government in the early days of the epidemic can effectively control the spread of the epidemic. Combined with the transmission frequency equation of COVID-19 epidemic, measures taken by the government are analyzed. The influence of each measure on the frequency variable is judged and then the influence on the spread of the epidemic is obtained. Finally, based on the above analysis, the government is advised to adhere to the principles of scientific, initiative and flexibility when facing major epidemics.

## Introduction

An outbreak of COVID-19 occurred in Wuhan, China in December 2019. It’s an acute infectious pneumonia. Prior to this, similar acute infectious diseases such as severe acute respiratory syndrome (SARS), avian influenza, H1N1 influenza, etc. had outbreak in many parts of the world due to their high infectivity. The outbreak of major epidemics are often influenced by both human and natural factors. Major infectious diseases spread rapidly and widely, and some viruses have the characteristics of strong climbing ability and human-to-human transmission. If the government and the public do not take measures to prevent and control the epidemic in time, very serious consequences for social economy and public health would happen. Therefore, from the perspective of social and economic stability and the public’s safety, the government’ prevention and control measures and the public’s attitude of cooperation are significant to the control of the epidemic.

With the outbreak of major infectious diseases, some scholars have studied the epidemic prevention and control from the perspective of the government intervention and the public behavior. Scholars point out that government intervention measures [1–5], public knowledge of epidemic prevention and control [6], cautious attitude towards epidemic [7], trust in information [8], and risk awareness [9] will affect the control of epidemic. Moreover, the effectiveness of the new management model of the government [10] and how to control the spread of infectious diseases effectively from the perspective of law enforcement [11, 12] have been studied. From the perspective of the government and the public, the above scholars have analyzed and summarized the prevention and control of the epidemic by using qualitative methods at the macro level, providing experience for the study of the COVID-19 epidemic. In addition, some scholars have used SIR model [9], SIRS model [13], SI model [14, 15], propagation dynamics model [16, 17], generalized stochastic Petri net model and equivalent Markov chain model [18], high-dimensional dynamic model [19] and other mathematical tools to analyze major public health events. Li et al. [20] studied an epidemic model with both diffusion and migration. Through mathematical analysis and numerical simulation, it is found that the model presents a typical moving mode. Jin et al. [21] constructed an evolutionary game model based on the credibility of the government and the trust of the people. Zu et al. [22] evaluated the effectiveness of border quarantine, isolation, treatment and other measures to prevent influenza by establishing a computer epidemic model in Beijing. Sun et al. [23] built a disease transmission control model based on the spread force, and simulated the spread process of the disease to build an infection tree, and proposed the target immune algorithm to provide support for the emergency management of disease control. Mei et al. [24] introduced spatial information, urban traffic, individual behavior and other factors into the urban air infectious disease diffusion model, and verified the effectiveness of the model by simulating the spread of infectious diseases with a simulation system. The above studies provide a reference for this study by using the mathematical model to analyze the epidemic situation, but the relevant models still need to be further optimized.

Since the global outbreak of COVID-19 in 2020, some scholars have researched the epidemic from the aspects of clinical characteristics and bio-informatics. Zhu et al. [25] reviewed the clinical and chest imaging features of COVID-19 and compared them with other infectious and non-infectious diffuse lung diseases to find clues for the diagnosis of COVID-19 to ensure accurate diagnosis and treatment. Chan et al. [26] identified a novel human corona virus based on RNA from two cases of pneumonia in Wuhan in 2019. Chan et al. [27] carried out bioinformatics analysis on the viral genome of a patient infected with COVID-19 and compared it with other corona virus genomes to provide a basis for studying the pathogenesis, optimal diagnosis, antiviral and vaccination strategies of the disease. Yang et al. [28] made a comparative analysis of the SARS pandemic and the 2019-nCoV. Combined with the lessons of SARS epidemic, the nucleic acid sequence can be determined and isolation strategy can be adopted quickly. The above studies mainly compare and analyze the COVID-19 with other pulmonary diseases. There are few studies involving evolutionary games and government intervention in COVID-19.

To sum up, the above studies mainly focus on qualitative analysis or quantitative analysis, but lack of a good integration. Moreover, there are few studies on modeling and forecasting the development of COVID-19 using evolutionary game from the perspective of the government. In view of these, this study fully considers the social economic problems and the natural transmission law involved in the epidemic, and establishes the transmission frequency equation of COVID-19 epidemic based on the evolutionary game theory and infectious disease diffusion model. Through the analysis of the early data of COVID-19, the validity of the model is verified, and on the basis of this, the impacts of the government’s measures on the epidemic control are analyzed.

## Materials and methods

### Analysis of COVID-19 epidemic evolution game model

#### Model hypothesis

Evolutionary game theory is a theory combining game theory analysis and dynamic evolutionary process analysis. Evolutionary game theory is originated from the theory of biological evolution. It successfully explains some phenomena in the process of biological evolution, abandons the assumption that game theory is completely rational and thinks that human beings usually achieve the balance of game through trial and error. At the same time, the theory can better analyze and solve the influencing factors of social habits, norms, institution and the problems in the process of spontaneous evolution. The development of the epidemic situation makes the government and the public’s awareness of the epidemic situation in a constantly changing process, and the response strategies adopted are also in the process of dynamic adjustment. In this dynamic process, the choice of subject strategy conforms to the "limited rationality" hypothesis that the participants of evolutionary game adopt imitation behavior. And the key of evolutionary game analysis is to determine the mode of learning and strategy adjustment of the government and the public during the development of the epidemic. This paper studies the dynamic evolutionary process of the two players’ repeated game over time and reach a certain stable state.

Based on evolutionary game theory, the interaction between the government and the public will inevitably occur during the prevention and control of major epidemics. The interaction between the two subjects is essentially a game process. In the face of major epidemics, assume that the strategy set of the government is “Emergency response” strategy (*U*) and “Laissez-faire” strategy(*I*), that is, *J*_1_ = {*U*, *I*}. The strategy set of the public is "Active isolation" strategy (*C*) and "Arbitrary flow" strategy(*F*), that is, *J*_2_ = {*C*, *F*}. The "Emergency response" strategy mainly refers to a series of measures taken by the government to actively respond to the epidemic situation, such as large-scale screening and isolation for close contacts and suspected exposed persons of COVID-19, targeted treatment, strict epidemic reporting, strengthening the management of medical institutions, and doing a good job in material support. The "Laissez-faire" strategy means that the government is indifferent to the COVID-19 epidemic. The "Active isolation" strategy refers to that the public complies with the relevant regulations on epidemic prevention and control, actively cooperate with the relevant personnel, and actively do self-protection work, etc. The "Arbitrary flow" strategy means that confirmed patients, suspected patients and close contacts ignore the prevention and control regulations and do not actively cooperate with treatment. At the same time, this strategy also refers to those patients who are still in the incubation period to enter the densely populated places randomly, resulting in the spread of COVID-19.

Based on the assumptions of the government’s strategies and the public’s strategies, four different game strategy combinations will appear during the interaction between these two subjects, namely (Emergency response, Active Isolation), (Laissez-faire, Arbitrary Flow),(Emergency Response, Arbitrary Flow),(Laissez-faire, Active Isolation).

Case 1: When faced with COVID-19, the government will combine the experience of previous major health emergencies to adopt the strategy of emergency response (*U*), and the cost of its active prevention and control of the epidemic situation is *c*. At this time, the cost caused by the public adopting active isolation strategy (C) is *a*.
Case 2: In the early days of the epidemic, the government and the public knew little about the COVID-19. Both of them may adopt evasion strategies. When the government adopts the strategy of laissez-faire (*I*) and the public adopts the strategy of arbitrary mobility (*F*). The probability of the spread of the epidemic becoming larger is *p*_1_. At this time, the economic loss caused by the epidemic to society is *L*. Therefore, when the government and the public take evasive actions, the social losses are *p*_1_∙*L*. The social losses caused by the large-scale spread of the epidemic are much higher than the prevention and control costs of the government, that is, *p*_1_∙*L* > *c*. The social losses of the spread of COVID-19 from arbitrary travel by confirmed or suspected COVID-19 patients have a negative externality. Therefore, the individual will bear part of the corresponding losses $\frac{p_{1}∙L}{n},\;|a|>|\frac{p_{1}∙L}{n}|$.
Case 3: Considering account political and economic factors and previous experience in fighting the epidemic, the government has taken a priority over the public in adopting strategies in the face of the epidemic. After the COVID-19 is found, even if the public adopts the strategy of arbitrary mobility (*F*), the government will adopt the strategy of emergency response (*U*). At this time, the probability of large-scale spread of COVID-19 is reduced to *p*_2_, *p*_1_ > *p*_2_. During the period of the epidemic prevention and control, if the public fails to comply with relevant laws and regulations and causes the expansion of COVID-19 infection scale, they will be severely punished. The corresponding punishment limit is *b*, and *b>a*.
Case 4: If the government adopts a laissez-faire strategy (*I*) in the face of an epidemic, the cost of prevention and control is zero. In the face of the epidemic, the public takes the initiative to understand the current situation of the epidemic and adopts the active isolation strategy (C) for their health and safety. The cost caused by this strategy is *a*.

It can be seen from the game matrix between the government and the public in major public health emergencies (Table 1). The best response strategy in the prevention and control of COVID-19 is Emergency Response (*U*), Active Isolation (*C*)), which is the perfect equilibrium path of sub-game.

**Table 1:** The game matrix between the government and the public in major public health emergencies.

**Strategy Choice**		**Public**	
		Active Isolation (*C*)	Arbitrary Flow (*F*)
**Government**	Emergency Response (*U*)	(−*c*, −*a*)	$\left(-c-p_{2}∙L,\;-b-\frac{p_{2}∙L}{n}\right)$
	Laissez-faire (*I*)	(0, −*a*)	$\left(-p_{1}∙L,\;-\frac{p_{1}∙L}{n}\right)$

Based on evolutionary game theory and combining the four strategy combinations of the government and the public in the face of the major epidemic, the payoff matrix of the game between the two sides is obtained.

#### Replication dynamics equation and infectious disease SI equation

The object of evolutionary game theory is a group that evolves over time. This theory no longer models the game as a super rational party, but illustrates the dynamic process of group evolution under the condition of group bounded rationality and explains why and how the group reaches this state. Among them, the replication dynamic model is the most common kind of learning model [29]. When applying the evolutionary game replication dynamic model to analyze social and economic system problems, the corresponding actions of different strategies are observed in different ways, and some strategic behaviors are discovered and learned. Therefore, Sethi proposed a generalized replication dynamic model to solve this problem [30].

In this model, the time *t* is evenly divided into several fixed time cycles of length *ρ*, and the individuals modify the strategy selection in each fixed interval. Randomly select a number of individuals in a large group and observe their income and behavior performance. The population proportion *θ*_*j*_ is used to express the probability that the individuals adopting strategy *j* are selected. If those individuals who adopt the strategy *j* are selected, *φ*_*j*_ represents the probability that the relevant benefits and behavior performance of the individual’s strategy are detected. If the individuals who adopt strategy *i* observe that their income is lower than the income of the individuals who adopt strategy *j*, then the probability of turning to adopt strategy *j* is proportional to the income difference (*ω*_*j*_ − *ω*_*i*_) between the strategies, after proper normalization of income, the probability is (*ω*_*j*_ − *ω*_*i*_). Therefore, in a fixed time interval of *ρ*, the proportion of individuals who choose strategy *i* turn to choose strategy *j* is $p_{i}^{j}$:

pij={ρφj(ωj-ωi)θj,ifωj>ωi;0,eles (1)

Define the collection:

$$W_{i}(\theta )=\{j\in I\;|\omega_{j}(\theta )>\omega_{i}(\theta )\}$$ (2)

Eq (2) includes all strategies whose incomes higher than strategy *i* when the population proportion distribution is *θ*(*t*).

The proportion of population in a fixed time interval *(t* + *ρ)* is:

$$\theta_{i}(t+\rho )=\theta_{i}(t)+\sum_{j{\notin W}_{i}(\theta )}\rho \varphi_{i}(\omega_{i}-\omega_{j})\theta_{i}(t)\theta_{j}(t)-\sum_{j{\in W}_{i}(\theta )}\rho \varphi_{j}(\omega_{j}-\omega_{i})\theta_{j}(t)\theta_{i}(t)$$ (3)

When the fixed time interval closes to zero, i.e. take the limit ρ→0, the general dynamic model of replication is as follows:

$$\frac{d\theta_{i}(t)}{dt}=\theta_{i}(t)∙[\varphi_{i}∙\sum_{j{\notin W}_{i}(\theta )}\left(\omega_{i}-\omega_{j}){∙\theta }_{j}(t\right)-\sum_{j{\in W}_{i}(\theta )}\varphi_{j}∙(\omega_{j}-\omega_{i}){∙\theta }_{j}(t)]$$ (4)

At the early days of the epidemic, the government and the public knew little about the pathogenic mechanism, transmission route and protective measures of the COVID-19. Human beings have the psychology of conformity and imitation in the period of major outbreaks. When the COVID-19 occurred, Academician Nanshan Zhong of China went to Wuhan to investigate and found that the COVID-19 had the risk of human-to-human transmission. Similar to SARS and influenza a (H1N1), the key to the prevention and control of COVID-19 is to control the human-to-human transmission. Therefore, isolation prevention and control measures are important to control the large-scale spread of the epidemic. Then, the spread of the epidemic can be indirectly expressed by the population proportion growth rate $\frac{d\theta_{F}(t)}{dt}$ of the public choosing arbitrary flow strategy (*F*):

$$\frac{d\theta_{F}(t)}{dt}=\theta_{F}(t)∙\varphi_{F}∙\sum_{F\in I}\left(\omega_{F}-\omega_{C}){∙\theta }_{C}(t\right)$$ (5)

Arranged:

$$\frac{d\theta_{F}(t)}{dt}=\theta_{F}(t)∙(1-\theta_{F})∙\varphi_{F}(\omega_{F}-\omega_{C})$$ (6)

It is worth noting that Eq (6) is similar to the SI equation for early study of infectious diseases:

$$\frac{dI(t)}{dt}=\eta ∙I(t)∙(1-I(t))$$ (7)

By comparing the SI model (7) with the general replication dynamic model of COVID-19 (6), it can be found that *η* in the SI model is defined as the number of healthy people infected by each patient per day per unit time, that is, the infection rate. The infection rate *η* depends on the income difference (*ω*_*F*_ − *ω*_*C*_) of different strategies adopted by the government and the public in the evolutionary game process and the observability *φ*_*F*_ of the action strategy, then, *η* = *φ*_*F*_ (*ω*_*F*_ − *ω*_*C*_). Thus, the transmission paths of SI model of infectious disease diffusion are enriched by the evolutionary game model of COVID-19 outbreak. Assuming that the total population of the society is M, and the number of the public adopting arbitrary flow strategy is N, it can be obtained by substituting them into Eq (6):

$$\frac{dN}{dt}=N∙(M-N)∙(\frac{\varphi_{F}}{M})(\omega_{F}-\omega_{C})$$ (8)

After separating the variables, integrate both sides, let *N*(0) = *N*_0_, the transmission frequency equation of COVID-19 epidemic is as follows:

$$N(t)=\frac{1}{\frac{1}{M}+(\frac{1}{N_{0}}-\frac{1}{M})∙e^{-\beta t∙M}}among,\beta =\frac{\varphi_{F}}{M}∙(\omega_{F}-\omega_{C})$$ (9)

Eq (9) indicates the change in the number of socially infected people when the government adopts the emergency response strategy and the public always chooses the arbitrary mobility strategy. From the game matrix between the government and the public in major public health emergencies (Table 1), it can be seen that in the case of the government’s emergency response to the COVID-19 epidemic, the payment for the public to adopt the active isolation strategy is *ω*_*C*_ = −*a*, while the payment for the public to adopt the arbitrary mobility strategy is $\omega_{F}=-b-\frac{p_{2}*L}{n}$. By substituting the two payments into Eq (9), it can be obtained that when the government responds to the COVID-19 epidemic urgently, the transmission frequency equation of the COVID-19 epidemic is:

$$N(t)=\frac{1}{\frac{1}{M}+(\frac{1}{N_{0}}-\frac{1}{M})∙e^{-\varphi_{F}∙(a-b-\frac{p_{2}*L}{n})t}}$$ (10)

### Test based on the epidemic data of COVID-19

Different epidemic evolution scenarios are assumed in this section. And the upper limit of the spread of the COVID-19 epidemic *M* is set according to the cumulative daily confirmed cases in China published by the Chinese Health Commission and the cumulative daily confirmed cases in the UK published by the websites of WHO and Hopkins University. The frequency fitting equation of cumulative confirmed cases of COVID-19 in different evolution scenarios are obtained through logistic curve fitting by SPSS. The Logistic model in curve estimation in SPSS is:

$$N(x)=\frac{1}{\frac{1}{v}+b_{0}∙b_{1}^{x}}$$ (11)

By comparing Eq (10) with Eq (11), it can be seen that:

$$v=M,\;b_{0}=(\frac{1}{N_{0}}-\frac{1}{M}),\;b_{1}=e^{-\beta }$$ (12)

#### Evolution scenario 1: The evolution of the epidemic under the laissez-faire strategy of the government in the early days of the COVID-19

If the government does not take the emergency response strategy in the early days of the COVID-19, and the public moves arbitrarily, it will lead to the further spread of the epidemic and then threaten human health and social and economic development. This scenario is regarded as the evolution of the epidemic when the government adopts the laissez-faire strategy in the early days of the COVID-19. For illustration purposes, this scenario uses the cumulative confirmed cases of UK from January 31 to April 19, 2020. And relevant data are divided into a training set and a test set according to 0.8:0.2. The data on May 10 is selected as the upper limit, M = 216526, and the frequency fitting equation for the cumulative confirmed cases is:

$$N(t)=\frac{1}{1/216526+2.179∙0.840^{t}}$$ (13)

**Fig 1:**
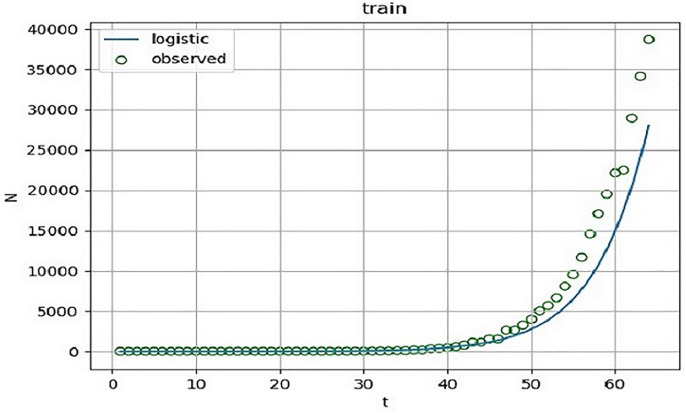
Logistic curve fitting diagram of cumulative confirmed cases of COVID-19. Taking the cumulative confirmed cases of COVID-19 in the UK from January 31 to April 3, 2020as a training set, and setting the upper limit M = 216526. And SPSS is used to fit the Logistic curve of the daily cumulative number of confirmed cases in this period.

**Fig 2:**
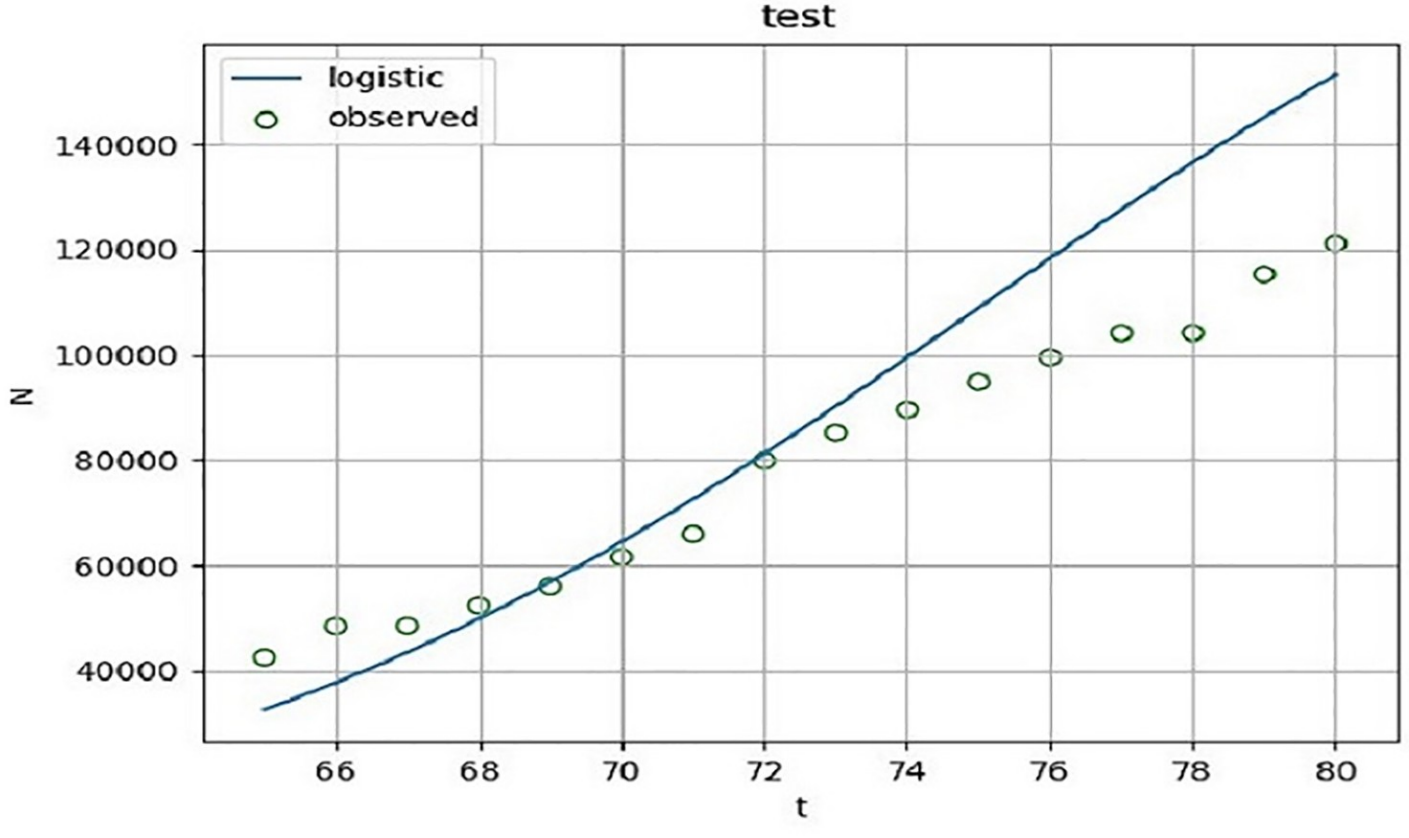
Logistic curve fitting diagram of cumulative confirmed cases of COVID-19. The cumulative confirmed cases of COVID-19 in the UK from April 4 to April 19, 2020 are used as a test set. SPSS is used to conduct Logistic curve fitting for the daily accumulative confirmed number in this period to evaluate the effectiveness of the model.

According to the fitting results of the training set (Fig 1 and Table 2), the determinable coefficient of logistic regression is 0.823. In other words, as of April 3, 2020, more than 82.3% of the changes of the cumulative confirmed cases can be explained by the above model with a high degree of fitting. The F statistic is 287.875, P < 0.0001, and the model is notable. In order to ensure the validity of the model, the model is predicted. According to the fitting results of the test set (Fig 2 and Table 2), the determinable coefficient of logistic regression is 0.849. In other words, as of April 19, 2020, more than 84.9% of the changes of the cumulative confirmed cases can be explained by the above model with a high degree of fitting. The F statistic is 78.426, p<0.0001, the model is notable.

**Table 2:** Estimated parameters of cumulative confirmed cases of COVID-19.

Equation	R Square	F	Sig.	b_0_	b_1_
**Training set**	0.823	287.875	0.000	2.179	0.840
**Test set**	0.849	78.426	0.000	2.179	0.840

Curve fitting analysis is performed on the cumulative number of confirmed COVID-19 patients in evolutionary scenario 1 to obtain relevant parameters, which provide certain basis for the illustration of the effectiveness of the model.

#### Evolution scenario 2: The evolution of the epidemic under the emergency response strategy of the government in the early days of the COVID-19

**Fig 3:**
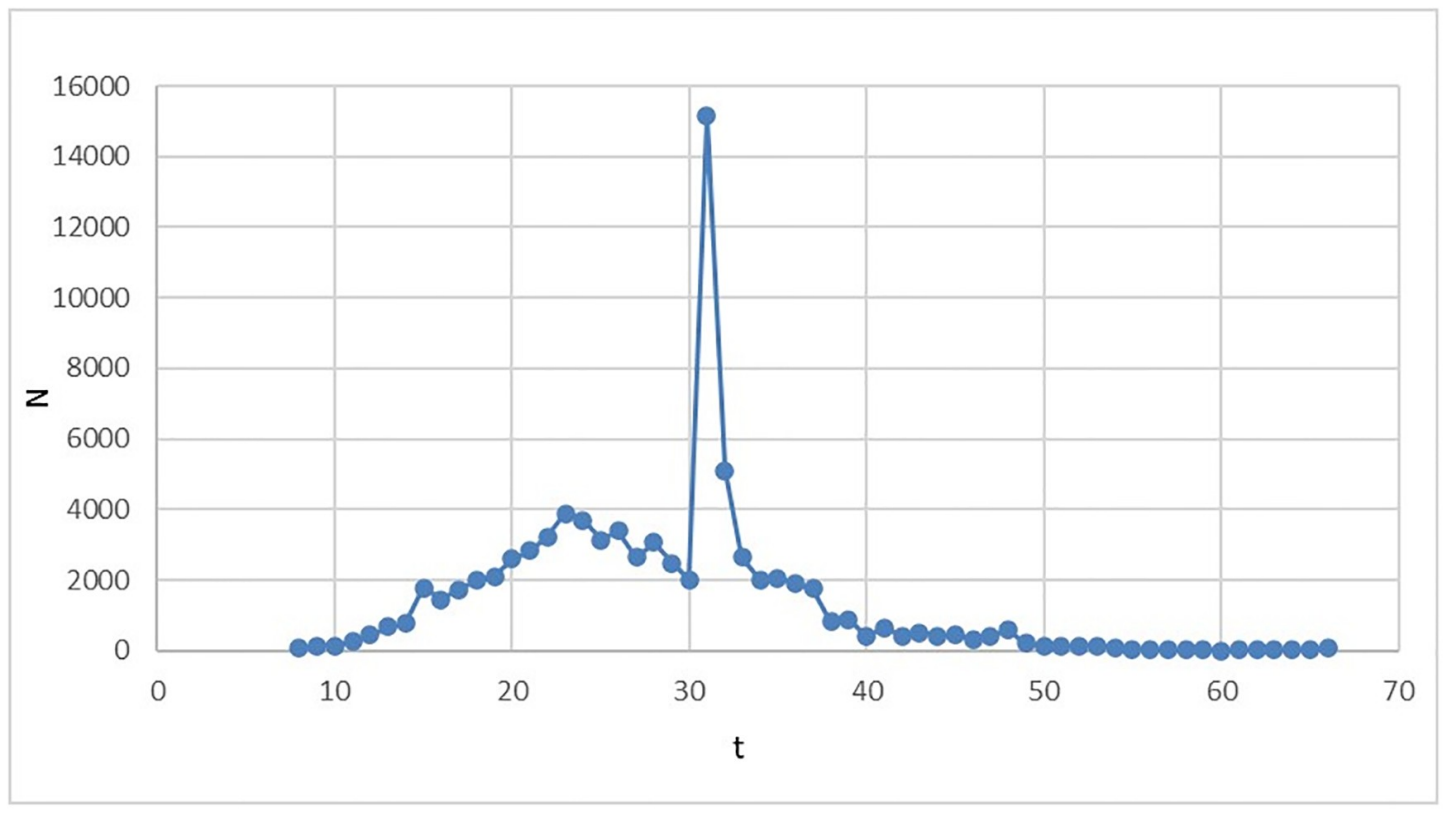
The number of new cases per day in China in 2020 (January 13, 2020 to March 18, 2020). The number of new COVID-19 cases per day in China during the period from January 13 to March 18, 2020 is made into a line chart, so that the evolution trend of new COVID-19 cases per day could be presented more intuitively.

In the early days of the COVID-19, the government will take emergency response measures actively, and with the development of the epidemic and the deepening understanding of the virus, the government will adjust relevant prevention and control strategies, which could control the large-scale spread of the epidemic. This scenario is regarded as the evolution of the epidemic when the government adopts the emergency response strategy in the early days of the epidemic. For illustration purposes, this scenario uses the cumulative confirmed cases of China from January 13 to February 21, 2020. And relevant data are divided into a training set and a test set according to 0.8:0.2. It can be seen from Fig 3 that the daily new cases tend to be flat at a lower level after February 21. Therefore, the training set takes the cumulative number of confirmed cases on that day as the upper limit of this evolutionary scenario, M = 76392. The fitting equation of the cumulative number of confirmed cases is as follows:

$$N(t)=\frac{1}{1/76392+0.034∙0.741^{t}}$$ (14)

**Fig 4:**
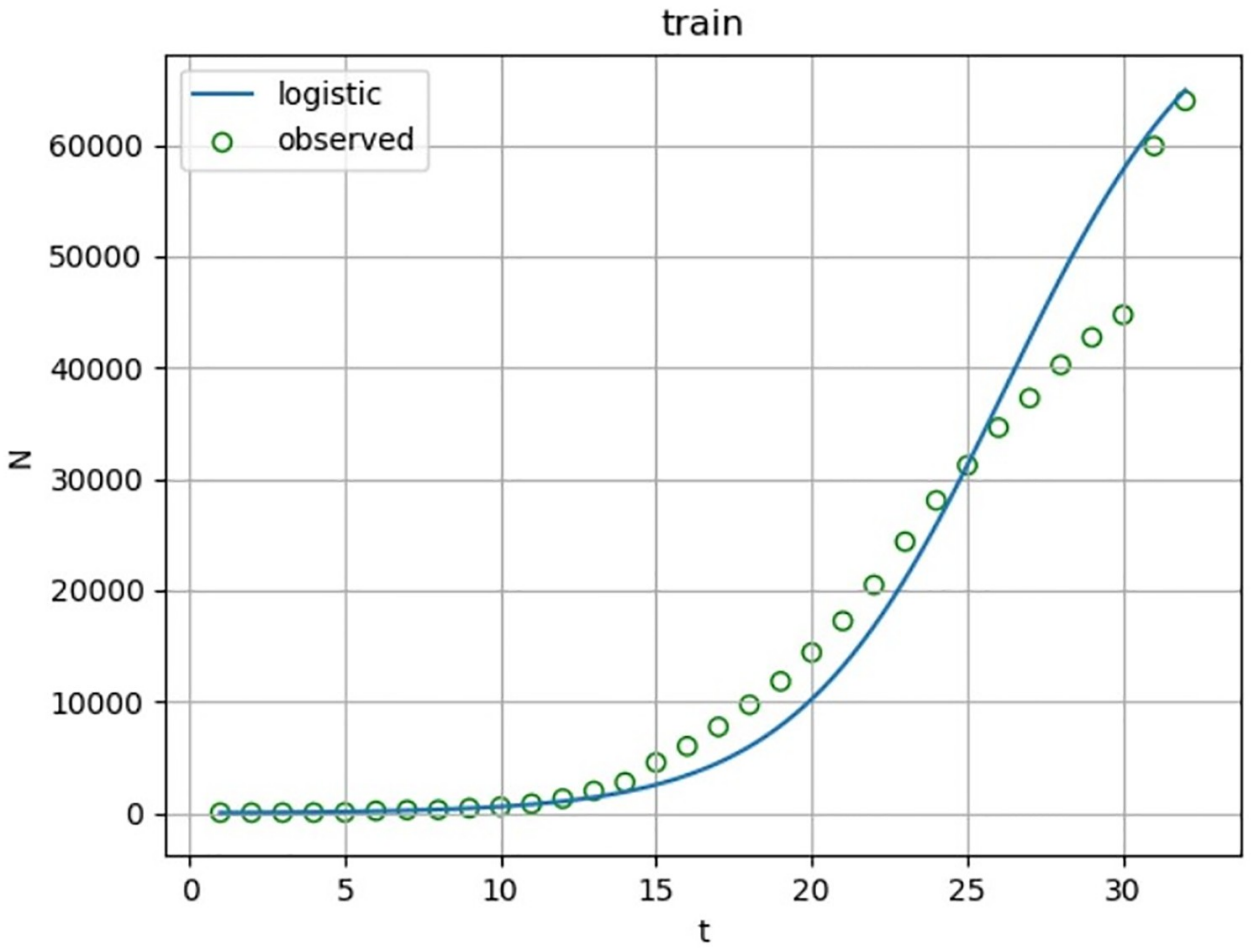
Logistic curve fitting diagram of cumulative confirmed cases of COVID-19. Taking the cumulative confirmed cases of COVID-19 in China from January 13 to February 13, 2020 as a training set, and setting the upper limit M = 76392. And SPSS is used to fit the Logistic curve of the daily cumulative number of confirmed cases in this period.

**Fig 5:**
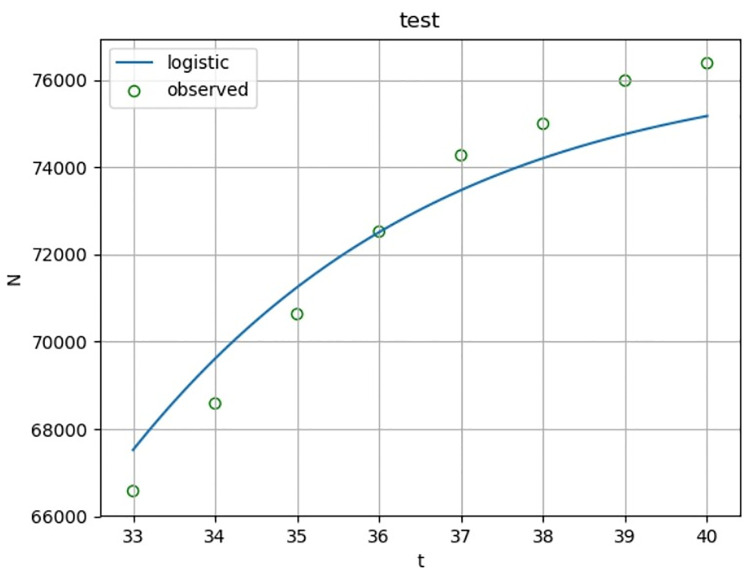
Logistic curve fitting diagram of cumulative confirmed cases of COVID-19. The cumulative confirmed cases of COVID-19 in China from February 14 to February 21, 2020 are used as a test set. SPSS is used to conduct. Logistic curve fitting for the daily accumulative confirmed number in this period to evaluate the effectiveness of the model.

According to the fitting results of the training set (Fig 4 and Table 3), the determination coefficient of Logistic regression is 0.966. In other words, as of February 13, 2020, more than 96.6% of the changes of the cumulative confirmed cases can be explained by the above model with a high degree of fitting. The F statistic is 859, p<0.0001, the model is notable. In order to ensure the validity of the model, the model is predicted. According to the fitting results of the test set (Fig 5 and Table 3), the Logistic regression of determination coefficient is 0.886. In other words, as of February 21, 2020, more than 88.6% of the changes of the cumulative confirmed cases can be explained by the above model with a high degree of fitting. The F statistic is 46.487, p = 0.0005, the model is notable.

**Table 3:** Estimated parameters of cumulative confirmed cases of COVID-19.

Equation	R Square	F	Sig.	*b*_0_	*b*_1_
**Training set**	0.966	859	0.000	0.034	0.741
**Test set**	0.886	46.487	0.005	0.034	0.741

Curve fitting analysis is performed on the cumulative number of confirmed COVID-19 patients in evolutionary scenario 2 to obtain relevant parameters, which provide certain basis for the illustration of the effectiveness of the model.

### Comparative analysis of scenario 1 and scenario 2

In evolutionary scenario 1, the target country began to gradually unseal due to the effective control the spread of COVID-19 on May 11, 2020. Then the first day of unsealing, i.e. t = 102, is brought into the fitting Eq (13), it can be found that the cumulative number of confirmed COVID-19 patients is 214612. In evolutionary scenario 2, the cumulative number of confirmed COVID-19 patients on the 102nd day is 76392. This indicates that the emergency response measures taken by the government in the early days of the COVID-19 can effectively control the spread of the epidemic.

In evolutionary scenario 2, according to the fitted Eq (14), the spread of COVID-19 reached its peak when t = 70, that is, the cumulative number of confirmed cases is 76392. However the cumulative number of confirmed cases in the target country still shows an upward trend on the 71st day. It indirectly indicates that the COVID-19 can be effectively controlled if the government could take timely emergency measures in the early days of the epidemic.

## Results and discussions

The widespread spread of the COVID-19 will have a huge negative impact on society. It is beneficial to human health and social development for the government to be vigilant and do a good job in epidemic prevention and control. With the research and understanding of the virus, the government has implemented relevant prevention and control measures to control the expansion of the epidemic. The analysis of the relevant prevention and control programs and intervention behaviors of the governments not only of great significance to reduce the scale of the spread of the epidemic, but also has reference value for the future outbreak of major public health events. The influence of the government prevention and control measures on the speed of epidemic transmission can be judged by deducing and analyzing the relevant variables of the transmission frequency equation of COVID-19 epidemic.

Taking the first derivative of *φ*_*F*_ with respect to *N*(*t*), it can obtain $\frac{\partial N(t)}{\partial \varphi_{F}}>0$. Then *φ*_*F*_ is positively correlated with *N*(*t*). When the spread of epidemic *φ*_*F*_ decreases due to the reduction of social panic, the speed of epidemic transmission *N*(*t*) will also decrease. When $\varphi_{F}(a-b-\frac{p_{2}∙L}{n})t>0$, it can get $\frac{\partial N(t)}{\partial M}>0$, that is, *M* is positively correlated with *N*(*t*). When the spread of the epidemic *M* is reduced, the speed of epidemic transmission *N*(*t*) will also decrease. Taking the first derivative of *a* with respect to *N*(*t*), it can obtain $\frac{\partial N(t)}{\partial a}>0$. Then *a* is positively correlated with *N*(*t*). When the cost of the active isolation strategy for the public is reduced, the speed of epidemic transmission *N*(*t*) will also be decrease. Taking the first derivative of *b* with respect to *N*(*t*), it can obtain $\frac{\partial N(t)}{\partial b}<0$. Then *b* is negatively correlated with *N*(*t*). When the cost of the public not cooperating with prevention and control work is increased, the speed of epidemic transmission *N*(*t*) will decrease.

### Relevant measures to reduce the spread of epidemic *φ*_*F*_ due to social panic

In order to reduce the spread of the epidemic caused by social panic, the relevant measures adopted by the government include: clearly implementing designated hospitals, establishing sheltered hospitals, accurate prevention and control of COVID-19 in different regions at different levels, making authoritative information public the first time and implementing an epidemic day reporting system.

The designated hospitals are defined and the methods of "centralizing patients, experts, resources and treatment" are adopted to actively treat patients in order to improve the treatment rate and reduce mortality. Square cabin hospital plays an irreplaceable role in treatment, which is a new mode of responding to public health emergencies and major disasters to rapidly expand medical resources. Accurate prevention and control of COVID-19 refers to the division of regions into three levels of low risk, medium risk and high risk by government departments, and different prevention and control measures are taken for regions of different levels. By formulating differentiated prevention and control strategies to restore production order in an orderly manner, promote economic development and maintain social stability, it is helpful to reduce the panic caused by the development of the epidemic. In addition, one of the keys to fighting the COVID-19 is to know the actual situation and accurate data in a timely manner. Unclear epidemic situation will delay the best opportunity for decision-making. Responding positively to the public’s concerns will help create a favorable atmosphere for epidemic prevention and control and reduce public speculation about the epidemic. The implementation of the epidemic day reporting system means that the national and local health commissions release daily COVID-19 epidemic data, so that the public can have an objective understanding of the development of the epidemic. As a result, the probability of public panic caused by speculation is reduced, and then the prevention and control cost of the government is also reduced. It can be seen from the variation of variables in the frequency equation, these measures reduce the spread of epidemic *φ*_*F*_ caused by social panic, thus reduce the speed of epidemic transmission *N*(*t*).

### Relevant measures to reduce the spread range of the epidemic *M*

The government has reduced the spread of the epidemic range *M* by strengthening surveillance of the epidemic and epidemiological investigation of new cases, as well as strengthening case detection and screening. Strengthening the surveillance of epidemic and epidemiological investigation of new cases means that the government based on epidemiology, strengthen the prevention and control of key sites, carry out blanket search and grid management to control the large-scale spread of the epidemic. The public can find out whether patients are traveling with them by inputting the frequency, time and region of the trip through the method of intra-city inquiry. This measure makes full use of big data, cloud computing and other scientific means to monitor the epidemic. Continuously reduce the spread range of the epidemic *M*, thus reducing the spread speed *N(t)*. Strengthening case detection and screening means that disease control experts and relevant personnel strengthen the screening of cases to achieve "early detection, early isolation, early reporting, and early treatment". Screening of febrile patients aims to timely diagnosis and isolation of COVID-19 patients to prevent missed diagnosis and misdiagnosis of mild patients into severe patients. It can be seen from the variation of variables in the frequency equation, the prevention and control measure of strengthening case detection and screening has continuously narrowed the spread range *M*, reduced the prevention and control cost of the government, and reduced the speed of epidemic transmission *N*(*t*).

### Relevant measures to reduce the public’s active isolation cost *a*

The government has taken the following measures to reduce the cost of active isolation for the public: strengthening health education and prevention knowledge popularization, establishing hierarchical diagnosis and treatment system, and reducing the diagnosis and treatment fees for COVID-19 patients.

In order to strengthen the public’s understanding of the prevention and control measures of COVID-19, the government has made full use of radio, television, newspapers and Internet media to promote health education and prevention knowledge, and called on hospitals to carry out online consultation service of COVID-19. Through the popularization of prevention and control knowledge, this measure can improve the public’s awareness and prevention and control ability of the epidemic, effectively reduce the cost of active isolation of the public *a*, and thus reduce the speed of epidemic transmission *N*(*t*). The establishment of a hierarchical diagnosis and treatment system, as well as the medical expense deduction and medical insurance reimbursement guarantee mechanism for patients can minimize the probability of cross-infection, which is conducive to improving medical efficiency and eliminating the phenomenon that treatment is affected or abandoned due to cost. It can be seen from the variation of variables in the frequency equation, these measures can reduce the active isolation cost *a* of the public, so as to promote the choice of "Active isolation" strategy and reduce the speed of epidemic transmission *N*(*t*).

### Relevant measures to increase the cost of the public non-compliance with prevention and control *b*

Since the outbreak of COVID-19, the Chinese government has paid extensive attention to the hunting and eating of wildlife. At the 16th meeting of the Standing Committee of the 13th National People’s Congress in China, it was clearly pointed out that the illegal wildlife trade was comprehensively prohibited. Those who engage in illegal wildlife trade will be investigated for legal responsibility according to the law. During the epidemic prevention and control period, the government strengthened the supervision of the spread of false information. Behaviors that drive up prices and refuse to cooperate with related prevention and control work will be punished accordingly. It can be seen from the variation of variables in the frequency equation, it can be seen that these measures increase the cost of the inactive cooperation *b* of the public, promote the active cooperation of the public, and reduce the speed of epidemic transmission *N*(*t*).

## Conclusions

Considering the current situation of COVID-19 epidemic development and combining the evolutionary game theory and epidemic transmission model, some suggestions on epidemic prevention and control for the government are proposed:

1. Adhering to the scientific principle and implement the policy accurately. Both epidemiological studies of COVID-19 and policy adjustments based on the COVID-19 epidemic spread model fully demonstrate that the "scientific" principle will greatly enhance the government’s ability to intervene in the epidemic. Combined with the transmission frequency equation of COVID-19 epidemic and the government control and prevention measures, it can be found that the release of authoritative information, surveillance, epidemiological investigation of new cases, and the release of daily disease data can effectively reduce the speed of COVID-19 epidemic transmission. In addition, these measures can urge the public to adopt the strategy of "Active isolation", so that the epidemic can be controlled in the early days of the outbreak. Therefore, it shows that the scientific prevention and control measures and precise implementation of policies can improve the prevention and control ability of the government.
2. Adhering to the principle of initiative and flexibility is the key to the government’s response to the epidemic. COVID-19 has the characteristics of strong infectiousness. If epidemic is allowed to spread, it will cause direct harm to social economy and public health. Given the high threat, the government must intervene early and flexibly if the cases are found. For example, the government adopts measures such as active treatment, quarantine observation, and follow-up investigation to reduce the chance of virus mutation and spread. The development of COVID-19 can be controlled by classifying COVID-19 patients into different categories, carrying out precise prevention and control at different levels, adopting different punishment measures for those who violate regulations, and reducing the treatment costs of COVID-19 patients according to the medical insurance of different social groups and the affordability of families. The optimal solution of evolutionary game analysis can be concluded. "The government’s emergency response and the public’s active cooperation" also explains from the perspective of social economy that the government should follow the principle of "initiative and flexibility" in the response to COVID-19 epidemic, and always pay attention to the patients’ dynamics.

In this paper, the interaction strategies and the evolutionary game analysis of the actions taken by the government and the public in the early days of the epidemic are incorporated into the natural transmission mechanism model of the epidemic. And then the transmission frequency equations of COVID-19 epidemic is established. Based on the cumulative number of confirmed cases of COVID-19 in the UK and China, different evolutionary scenarios of the epidemic are set up. Moreover, SPSS is used to conduct Logistic curve fitting, and the frequency fitting equations of cumulative confirmed cases under different evolutionary scenarios are obtained respectively. The results show that the emergency response strategy adopted by the government in the early days of the epidemic could effectively control the spread of the epidemic. Finally, combined with the transmission frequency equation of COVID-19 epidemic, measures taken by the government are analyzed to judge the impacts of various measures on the spread of the epidemic. Based on the above analysis, suggestions are proposed for the government to adhere to the principles of science, initiative and flexibility in the face of major epidemics. But the socio-economic evolution model and the natural transmission mechanism model adopted in this paper cannot fully explain the COVID-19 epidemic, and the theoretical model still needs to be further optimized to promote the study of the epidemic.

## Supporting information

S1 File (XLSX)

S2 File (XLSX)
